# Phase III Soft Tissue Sarcoma Trials: Success or Failure?

**DOI:** 10.1007/s11864-017-0457-1

**Published:** 2017-03-23

**Authors:** Alexander T. J. Lee, Seth M. Pollack, Paul Huang, Robin L. Jones

**Affiliations:** 1 Sarcoma Unit, The Royal Marsden NHS Foundation Trust, London, UK; 2 Division of Cancer Biology, The Institute of Cancer Research, London, SW3 6JB UK; 3 Fred Hutchinson Cancer Research Center, Seattle, WA USA; 4 University of Washington, Seattle, WA USA; 5 Division of Clinical Studies, The Institute of Cancer Research, London, UK; 6 Sarcoma Unit, Royal Marsden Hospital, Fulham Road, London, SW3 6JJ UK

**Keywords:** Soft tissue sarcoma, Systemic therapy, Clinical trial design, Trial endpoints

## Abstract

Two recently reported phase III randomised control trials (RCTs) have resulted in the registration of two new systemic therapies for advanced soft tissue sarcoma. Both of these trials’ designs were informed by phase II data that guided the selection of sensitive STS diagnoses, enabling the demonstration of benefit in certain subtypes. A number of other phase III trials reported in the last 18 months have seemingly fit into a recurrent pattern of failure—promising efficacy signals in earlier phase studies being lost in the survival follow-up of large, highly heterogeneous cohorts. Greater effort is needed to identify histological and molecularly defined subgroups associated with differential treatment response in order to avoid the tremendous disappointment and loss of resources associated with a failed phase III trial. Additionally, improvements in available treatment of advanced STS have underpinned a prolongation in overall survival (OS). Consequently, surrogate efficacy endpoints are of increasing importance to STS drug trials. Whilst progression-free survival (PFS) should arguably replace overall survival as the primary endpoint of choice in first-line studies, more work is required to provide definitive validation of surrogacy, as well as developing more sophisticated techniques of assessing radiological response and expanding the inclusion of quality-of-life-related endpoints.

## Introduction

Cytotoxic chemotherapy is the mainstay of treatment for advanced STS (Table 1). First introduced in the 1970s, doxorubicin was one of the first anticancer agents to consistently produce meaningful rates of response in advanced STS, and remains a standard of first-line therapy [2]. Over the ensuing years, many other cytotoxic agents have been trialled in this setting, with only a handful demonstrating meaningful efficacy in unselected STS populations [5–10]. The combination of doxorubicin with a second active agent has been investigated on a number of occasions—whilst these more intensive schedules have often produced favourable rates of response at the expense of higher levels of toxicity, no trial has shown evidence of prolonged survival with combination therapy [2, 3••]. The differential efficacy of some drugs in certain STS subtypes has been highlighted, such as the case of paclitaxel in angiosarcoma [22, 23]. Meanwhile, beyond the targeting of mutated KIT and PDGFRA in gastrointestinal stromal tumours, emergent understanding of underlying molecular pathology has informed certain therapeutic strategies in rarer STS subtypes [20, 21, 24, 25]. Additionally, the multi-targeted tyrosine kinase inhibitor pazopanib was approved in 2012 and remains the only molecularly targeted agent approved across many different STS subtypes [11••].

**Table 1:** Current standard systemic therapies for advanced STS

Drug	Type	Indication	NIH NCI Level of evidence [1]	References
Doxorubicin	Anthracycline	First line	3iiiDiv	[2]
Doxorubicin + ifosfamide	Anthracycline + alkylating agent	First line (esp. chemo-sensitive subtypes/ bulky disease)	1iiDiii	[3]
Doxorubicin + olarutumab	Anthracycline + anti-PDGFRα monoclonal antibody	First line	1iiA	[4•]
Ifosfamide	Alkylating agent	Second line	1iiDiv	[5–7]
Gemcitabine + dacarbazine	Nucleoside analogue + alkylating agent	Second line	1iiA	[8]
Gemcitabine + docetaxel	Nucleoside analogue + taxane	Second line (potentially first line in some subtypes	1iiDiii	[9, 10]
Pazopanib	Multi-targeted kinase inhibitor (activity against VEGFRs, PDGFRα, FGFR1, KIT)	Second line and beyond in non-adipocytic STS	1iDiii	[11••]
Eribulin	Microtubule inhibitor	Second line (after anthracycline)	1iiA	[12••]
Trabectedin	DNA minor groove binder	Second to third line (after anthracycline and ifosfamide)	1iiDiii	[13••, 14]
Dacarbazine	Alkylating agent	Third line	3iiiDiv	[15, 16]
Liposomal doxorubicin	Anthracycline	Kaposi and angiosarcoma Substitute for doxorubicin in most STS	3iiiDiv	[17–19]
Sirolimus	mTOR inhibitor	Malignant PEComa	3iiiDiv	[20, 21]
Paclitaxel	Taxane	Kaposi and angiosarcoma	3iiiDiv	[17, 22, 23]
Crizotinib	Multi-targeted kinase inhibitor (activity against ALK, ROS1, MET)	Inflammatory myofibroblastic tumour	3iiiDiv	[24]
Imatinib	Multi-targeted kinase inhibitor (activity against KIT, PDGFRA, BCR-ABL)	Dermatofibrosarcoma protuberans	3iiDiv	[25]

The rarity and heterogeneity of STS pose a recurrent challenge to the design and conduct of adequately powered phase III drug trials. Although two recently reported studies have successfully led to new drug approvals, the majority of phase III trials have failed to result in new treatment registrations. In this review, we aim to provide an overview of recently reported phase III drug trials in advanced STS (summarised in Table 2) and examine the factors in trial design that may have contributed to their respective success or failure.

**Table 2:** Summary of recently published advanced STS phase III drug trials

Trial ID	Investigative arm	Control arm	Duration of treatment	Key eligibility criteria	Number	Median OS	Median PFS	ORR	DCR/CBR	Toxicity	QoL	Outcome
NCT01327885 [12••]	*Eribulin* (1.4 mg/m^2^ IV day 1 + 8) every 3 weeks	*Dacarbazine* (850–1200 mg/m^2^ IV day 1) every 3 weeks	Until disease progression	LPS or LMS^b^ Grade 2–3 ≥2 prior lines (inc anthracycline) PS0-2	452	13.5 vs 11.5 m (HR 0.77; 95% CI 0.62–0.95; *p* = 0.0169)^c^	2.6 vs 2.6 m (HR 0.88; 95% CI 0.71–1.09; *p* = 0.23)	4 vs 5%	6w DCR: 56 vs 53%	Excess G3–4 neutropaenia with eribulin Excess G3–4 anaemia + thrombocytopaenia with dacarbazine	In change of QoL between study arms	US and European approval for eribulin in LPS
NCT01168791 (PICASSO III) [26•]	*Doxorubicin* (75 mg/m^2^ IV day 1) plus *palifosfamide* (150 mg/m^2^ IV day 1–3) every 3 weeks	*Doxorubicin* (75 mg/m^2^ IV day 1) plus *placebo* every 3 weeks	Up to 6 cycles	Mixed STS^a^ No prior chemo except adjuvant GemTax PS0-2	447	15.9 vs 16.9 m (HR 1.05; 95% CI0.79–1.39; *p* = 0.74) **NB** after 59% target OS events)	6.0 vs 5.2 m (HR0.86; 95%CI 0.68–1.08; *p* = 0.19)^c^	28.3 vs 19.9%	12w CBR: 51.8 vs 41.2% (*p* = 0.02)	Excess febrile neutropaenia in palifosfamide arm	Not reported	Discontinued development of palifosfamide in STS
NCT01012297 (GOG-0250) [27]	*Gemcitabine* (900 mg/m^2^ IV day 1 + 8) plus *docetaxel* (75 mg/m^2^ IV day 8) plus *bevacizumab* (15 mg/kg IV day 1) every 3 weeks	*Gemcitabine* (900 mg/m^2^ IV day 1 + 8) plus *docetaxel* (75 mg/m^2^IV day 8) plus *placebo* every 3 weeks	Until disease progression	Uterine LMS^b^ No prior chemo PS0-2	107	OS 23.3 vs 26.9 m (HR 1.07; 95% CI 0.63–1.81; *p* = 0.81)	PFS 4.2 vs 6.2 m (HR 1.12; 95% CI 0.74–1.7; *p* = 0.58)	35.8 vs 31.5%	6w DCR 67.8 vs 62.5%	No significant difference in toxicity	Not collected	Little evidence of bevacizumab efficacy in any STS
NCT01343277 [13••]	*Trabectedin* (1.5 mg/m^2^IVI over 24 h day 1) every 3 weeks	*Dacarbazine* (1000 mg/m^2^ IV day1) every 3 weeks	Until disease progression	LPS or LMS Prior anthracycline + ifosfamide/ other chemo PS0–1	518	12.4 vs 12.9 m, HR0.84; 95% CI NR)^c^ **NB** after 50% of target OS events	4.2 vs 1.5 m (HR 0.55; 95% CI 0.44–0.70; *p* < 0.001)	9.9 vs 6.9%	18w CBR 34 vs 19%	Excess G3–4 neutropaenia and transaminitis in trabectedin arm.	Not collected	US approval of trabectedin for LPS and LMS (already available elsewhere)
NCT02672527 (T-SAR)^d^ [14]	*Trabectedin* (1.5 mg/m^2^ IVI over 24 h day 1) every 3 weeks	*Best supportive care*	Until disease progression	Mixed STS^a,b^ ≥1 prior lines (inc anthracycline) PS0–1	103	Not reported	3.1 vs 1.5 m (HR 0.39, 95% CI 0.26–0.63, *p* < 0.0001)^c^	Not reported	Not reported	Not reported	Not reported	Further evidence of efficacy in general STS population
ISRCTN07742377 (GEDDIS)^d^ [28]	*Gemcitabine* (675 mg/m^2^ IV day 1 + 8) plus *docetaxel* (75 mg/m^2^ IV day 8) every 3 weeks	*Doxorubicin* (75 mg/m^2^ IV day 1) every 3 weeks	Up to 6 cycles	Mixed STS^a,b^ No prior chemo PS0–2	257	63 vs 71w (HR 1.07; 95% CI 0.77–1.49)	PFS 23 vs 24w (HR 1.28; 95% CI 0.98–1.67; *p* = 0.07) ^c^	Not reported	6w DCR 58.6 sv 65.9%	More dose interruption and treatment cessation due to toxicity in GemTax arm	Not reported	Reaffirmation of doxorubicin as standard first line therapy
NCT00699517 [29]	*Cisplatin* (75 mg/m^2^ IV day 1) plus *ombrabulin* (25 mg/m^2^ IV day 1) every 3 weeks	*Cisplatin* (75 mg/m^2^ IV day 1) plus *placebo* every 3 weeks	Until disease progression	Mixed STS^a^ Prior anthracycline and ifosfamide PS0–2	355	11.4 vs 9.3 m (HR0.85; 95% CI 0.67–1.09; *p* = 0.197	1.54 vs 1.41 m (HR 0.76; 95%CI 0.59–0.98; *p* = 0.030)^c^	ORR 4 vs 1%	6w DCR 47 vs 36%	Higher rates of G3–4 neutropaenia, thrombocytopaenia and impaired LVEF in ombrabulin arm	Not collected	Ombrabulin dropped from development in STS
NCT01440088 (SARC021) [30•]	*Doxorubicin* (75 mg/m^2^ IV day 1) plus *evofosfamide* (300 mg/m^2^ IV days 1 and 8) every 3 weeks	*Doxorubicin* (75 mg/m^2^ IV day 1) plus *placebo* every 3 weeks	Dox up to 6 cycles Evofos until disease progression	Mixed STS^a^ Grade 2–3 No prior chemo (adjuvant therapy permitted) PS0–1	640	18.4 vs 19 m, HR 1.06; 95%CI 0.88–1.29) ^c^	6.3 vs 6.0 m (HR0.85, 95% CI0.70–1.03, *p* = 0.099)	28.4 vs 18.3%	6w DCR = 73.2 vs 65.9%	Excess infection, febrile neutropaenia, fatigue, GI toxicity and fatigue in evofosfamide arm	Not collected	Evofosfamide dropped from development in STS

^a^Subtypes commonly excluded from mixed STS cohorts including alveolar soft part sarcoma, extraskeletal myxoid chondrosarcoma, rhabdomyosarcoma, GIST, dermatofibrosarcoma, Ewing’s sarcoma, mixed mesodermal tumours, clear cell sarcoma, osteosarcoma, Kaposi’s sarcoma

^b^Protocol requirement for demonstration of objective disease progression within 6 months prior to enrolment

^c^Primary outcome

^d^Currently reported in abstract form only

## Recent successes—phase III studies resulting in new drug registration

### Eribulin

Eribulin mesilate is a synthetic analogue of a compound derived from the marine sponge *Halichondria okadai*. Eribulin disrupts microtubule propagation, conferring anticancer effects that include suppression of cancer cell migration and invasion, induction of vascular remodelling and reversal of epithelial-mesenchymal transition. Eribulin is approved for use in pre-treated metastatic breast cancer and has demonstrated single agent activity in advanced non-small cell lung cancer [31, 32].

A phase II trial conducted by the European Organisation for Research and Treatment of Cancer (EORTC) identified leiomyosarcoma (LMS) and liposarcoma (LPS), the so-called L-sarcomas, as STS subtypes of apparent sensitivity to eribulin [33]. In this non-controlled study, four independent subtype-defined arms were treated with eribulin with a primary outcome of 12-week progression-free survival rate (12w PFS). After treatment of 115 patients, 12w PFS in the LMS and LPS arms were 31.6 and 46.9%, respectively, whilst the synovial sarcoma and heterogeneous ‘other STS’ strata failed to meet the pre-specified >30% 12w PFS efficacy cutoff (12w PFS 21.1 and 19.2%, respectively).

A subsequent open-label phase III RCT of eribulin limited recruitment to the apparently sensitive L-sarcomas subtypes [12••]. Patients with pre-treated advanced disease were randomised to receive either eribulin or dacarbazine therapy until disease progression, unacceptable toxicity or withdrawal of patient consent. After randomisation of 452 patients and a median follow-up of 31 months, a statistically significant improvement in overall survival in the eribulin arm was seen (HR0.77, 95% CI 0.62–0.95, *p* = 0.0169). No difference in response, disease control and quality of life endpoints were seen between the study arms, whilst median PFS of 2.6 months was seen in both arms. Tolerability was broadly equivalent between both arms, although an excess of grade 1–2 peripheral neuropathy (19 vs 4%) and grade 3–4 neutropaenia (35 vs 16%) was seen in the eribulin arm. In analysis of pre-specified subgroups, the LPS group received a pronounced OS benefit with eribulin (median OS 15.6 vs 8.4 m, HR 0.511; 95% CI 0.35–0.75), whilst patients with LMS had similar outcomes regardless of treatment allocation (median OS 12.8 vs 12.3 m, HR 0.93; 95% CI 0.71–1.20). A 1.2-m increase in median PFS with eribulin in the LPS group reached statistical significance (HR 0.52, 95% CI 0.35–0.78) but was notably dwarfed by the 7.2-m OS gain in this subgroup. The trial technically did not have sufficient statistical power to test for interaction between eribulin effect and histological subtype, but regulatory bodies and others have interpreted these results as demonstrating that eribulin has a survival benefit over dacarbazine in LPS only.

### Trabectedin

Trabectedin is a marine-derivative antineoplastic agent with anticancer effects believed to primarily relate to binding to the minor groove of DNA, resulting in inhibition of transcription, DNA repair and replication [34]. Additional effects of trabectedin may also include modulation of tumour microenvironment factors including microvasculature and tumour-infiltrating macrophages [35]. Trabectedin has been shown to have particular effect in translocation-associated sarcomas such as myxoid LPS, where preclinical research has shown that trabectedin can reverse the transcriptional reprogramming orchestrated by the pathognomonic *FUS-CHOP* fusion gene [36].

Following encouraging results from three non-comparative studies, the first randomised evidence of trabectedin efficacy in advanced STS was from a phase II study where patients with pre-treated L-sarcomas were randomly allocated to one of two different trabectedin schedules (1.5 mg/m^2^ over 24 h q3w vs. 0.58 mg/m^2^ over 2 h weekly for three consecutive weeks in a 4-week cycle) [37]. Although lacking a non-trabectedin comparator arm, the superior time-to-progression and PFS of the 24 h schedule compared to the weekly dosing arm and to EORTC-defined efficacy benchmarks led to European approval in 2007 for use of trabectedin in STS following anthracycline and ifosfamide. The efficacy of trabectedin in translocation-associated sarcomas was established by a Japanese randomised phase II trial, where trabectedin (24-h infusion) conferred a large PFS benefit compared to best supportive care alone (median PFS 5.6 vs 0.9 m; HR 0.07; 95% CI 0.03–0.16; *p* < 0.0001) [38••]. The Japanese trial followed an international phase III study comparing trabectedin to doxorubicin in a first-line setting. This earlier study closed early after interim analysis led to an impractical inflation of target sample size as a response to high rates of patient drop-out and lack of any large early efficacy signal [39].

Two recently reported phase III trials have provided definitive evidence of the efficacy of 24 h infusional trabectedin compared to non-trabectedin control in less selected STS populations. The earlier and larger of these recruited 518 patients with pre-treated L-sarcomas (approximately 75% LMS, 25% LPS) and randomised 2:1 to either trabectedin or dacarbazine [13••]. A statistically significant improvement in median PFS was seen with trabectedin (4.2 vs 1.5 months), whilst the significant improvement in clinical benefit rate (defined as maintained response or stable disease to at least 18 weeks—34 vs 19%; *p* < 0.001) but not objective response rate (9.9 vs 6.9%) highlighted the largely tumour static effect of trabectedin in a pre-treated trial cohort where >50% patients had experienced progressive disease as best response to their previous line of therapy. Interim analysis of the trial’s primary OS endpoint after 50% of target OS events had occurred showed no difference between treatment arms. Whilst follow-up continued to allow for currently unreported final OS analysis, the authors noted that the better-than-expected median OS of 12.9 months in the dacarbazine arm suggested that post-trial treatments may mask any evidence of OS benefit of trabectedin. This evidence of trabectedin effect led to US Food and Drug Administration (FDA) approval being granted for pre-treated L-sarcoma in November 2015.

The T-Sar trial, recently reported at the annual meeting of the European Society of Medical Oncology, was conducted by the French Sarcoma Group [14]. In this phase III trial, 107 patients who had received between 1 and 3 prior lines of systemic therapy for advanced sarcomas of mixed subtype were randomised to receive either trabectedin or best supportive care with optional post-progression crossover. A doubling of median PFS was seen in the trabectedin arm at interim efficacy reporting (3.0 vs 1.4 m, HR 0.40; 95% CI 0.26–0.63, *p* < 0.0001). A significant PFS advantage with trabectedin was seen in both L-sarcoma and non-L-sarcoma subgroups—the numerically greater effect size in the L-sarcoma group (HR 0.33 vs 0.49) was not powered for statistical comparison to the non-L-sarcoma group. Regardless, this trial provides the first randomised comparative data to support the efficacy of trabectedin in pre-treated patients across multiple STS subtypes.

## Recent phase III studies that failed to improve on standard of care

### Palifosfamide

Efforts to improve the efficacy of alkylating drugs in STS chemotherapy regimens whilst reducing ifosfamide-specific toxicities led to the development of palifosfamide, the active DNA-alkylating metabolite of ifosfamide. The use of palifosfamide avoids the production of the metabolic by-products that are thought to be responsible for ifosfamide-related encephalopathy, haemorrhagic cystitis and renal toxicity. Additionally, the more predictable pharmacokinetics of palifosfamide bolstered hopes of improved efficacy compared to ifosfamide.

Following randomised phase II data indicating the superiority of combination palifosfamide-doxorubicin compared to doxorubicin alone, PICASSO III was a double-blind, placebo-controlled phase III RCT aimed at demonstrating superior survival from using upfront anthracycline-alkylator combination [26•, 40]. Patients with untreated advanced STS of a broad range of subtypes were randomised 1:1 to treatment with doxorubicin combined with either palifosfamide or placebo to a maximum of six cycles. This design mirrored that of the earlier EORTC 62012 open-label trial, where in a mixed STS population with confirmed progressing disease, the combination of ifosfamide with doxorubicin failed to demonstrate a significant advantage in overall survival despite improvements secondary PFS and response outcomes [3••]. After treatment of 447 randomised patients in PICASSO III, no significant difference in PFS or OS were seen between palifosfamide and placebo-containing arms, despite improved rates of objective response and disease control with palifosfamide. In pre-planned subgroup analysis, there was no survival benefit with palifosfamide in any age or subtype-defined patient groups. In the overall cohort, higher rates of haematological toxicity and febrile neutropaenia were seen in the palifosfamide-containing arm. There were incidences of encephalopathy and haemorrhagic cystitis seen in the palifosfamide arm, but these were much lower than historically reported in ifosfamide-based studies. Due to the lack of difference in median PFS and OS between the doxorubicin alone and doxorubicin/palifosfamide arms, further development of this drug has been terminated.

### Evofosfamide

Evofosfamide (TH-302) is a prodrug that is converted under hypoxic conditions to an active DNA-alkylating metabolite. It has been predicted that preferential metabolic activation of evofosfamide in the relative hypoxia of the tumour bed would reduce systemic levels of toxic metabolites whilst enhancing efficacy relative to ifosfamide [41]. Following dose-finding phase I studies of evofosfamide in combination with doxorubicin, a single-arm phase II trial of the combination in first-line advanced STS reported encouraging rates of objective response, progression-free and overall survival [42, 43].

In the open-label SARC021 phase III trial, a cohort of 640 patients with untreated advanced STS that was unselected by subtype or any marker of tumour hypoxia were randomised to receive either doxorubicin alone or in combination with evofosfamide, with those not progressing on combination able to continue maintenance evofosfamide after completion of 6 cycles of doxorubicin-containing therapy [30•]. Similar to the PICASSO III trial, objective evidence of recent disease progression was not required prior to patient enrolment in SARC021–EORTC 62012 mandated progression within 6 weeks of commencing trial therapy. In SARC021, there was no significant difference between combination and single agent arms in progression-free survival (median PFS 6.3 vs 6.0 months, HR 0.85, 95% CI 0.70–1.03; *p* = 0.099) or overall survival (median OS 18.4 vs 19 months, HR 1.06; 95% CI 0.88–1.29). Despite protocol-mandated use of primary GCSF prophylaxis in the combination arm, higher rates of febrile neutropaenia were seen with doxorubicin-evofosfamide compared to doxorubicin alone (18.2 vs 11.0%), whilst greater levels of grade 3–4 fatigue, GI disturbance and nutritional disorder were also seen with combination treatment. These disappointing phase III results have led to the discontinuation of the development of evofosfamide in advanced STS.

### Gemcitabine and docetaxel

Whilst both gemcitabine, a nucleoside analogue, and docetaxel, a taxane, possess modest single agent activity in advanced STS, combination schedules of the two drugs produced response rates ranging between 14 and 53% in non-comparative phase II trials [44, 45]. The activity of gemcitabine-docetaxel regimens (GemTax) that employ optimised fixed-dose-rate administration of gemcitabine have been compared to gemcitabine alone in two separate randomised phase II studies in advanced STS. The earlier SARC002 trial employed a Bayesian adaptive model to randomise 122 patients with mixed advanced STS (60% GemTax, 40% gemcitabine alone) and found superiority of GemTax in terms of objective responses, clinical benefit rate, PFS and OS [9]. In contrast, the French study reported no improvement in outcomes with a similar GemTax schedule in LMS patients randomised through a more conventional statistical design [10]. Regardless, based on the cumulative evidence of activity, combination gemcitabine and docetaxel is established in the armamentarium in the majority of STS subtypes, particularly leiomyosarcoma and undifferentiated pleomorphic sarcoma.

In the GEDDIS trial, an open-label UK phase III trial reported at ASCO 2015, the efficacy, tolerability and safety of GemTax was compared to that of standard doxorubicin in the first-line treatment of intermediate-to-high grade advanced STS of unselected subtype [28]. After treatment of randomised 257 patients, there was no significant difference in the primary endpoint of 24-week progression-free survival rate between the doxorubicin and GemTax arms (46.1 vs 46.0%, respectively). The hazard ratio for PFS numerically favoured doxorubicin (HR 1.28, 95% CI 0.98–1.67, *p* = 0.07) whilst no significant difference in objective response rate and median overall survival was seen. Dose delays and toxicity-related discontinuation were higher with GemTax. No differences in efficacy outcome were reported between subtype-defined subgroups, where 27% of patients had uterine LMS and 56% were categorised as ‘other STS’. This result appears to confirm that for an unselected STS population, single agent doxorubicin should be the preferred first-line option, given greater tolerability and potentially favourable efficacy.

### Gemcitabine and docetaxel with bevacizumab

There has been little reported evidence to support a role for the anti-vascular endothelial growth factor (VEGF) monoclonal antibody bevacizumab in the treatment of STS. The combination of bevacizumab with doxorubicin in a phase II study in unselected STS subtypes failed to surpass efficacy levels expected for doxorubicin alone, with a safety signal that suggested an increase in cardiac toxicity [46]. Optimism for bevacizumab in STS of blood vessel origin was quelled by a randomised phase II trial that failed to demonstrate incremental efficacy from the addition of bevacizumab to standard paclitaxel chemotherapy [47].

In a recently reported placebo-controlled phase III RCT conducted in the USA, the addition of bevacizumab to GemTax backbone was compared to a control arm of GemTax-placebo in a subtype-selective protocol that specifically recruited advanced uterine LMS [27]. Following randomisation of 107 patients with chemo-naive advanced disease, numerically but non-significantly worse PFS and OS were seen in the bevacizumab arm (median PFS 4.2 vs 6.2 m, HR1.12, *p* = 0.58; median OS 26.9 vs 23.3, HR 1.07, *p* = 0.81) with no difference in ORR, CBR or median duration of response seen between trial arms. These results add further questions as to any potential role of bevacizumab in this and other STS subtypes, although the trial highlighted the feasibility of performing and completing subtype-selective phase III drug trials within 4 years of first patient recruitment.

### Ombrabulin with cisplatin

Ombrabulin is a combrestatin-type vascular disrupting agent with demonstrated single agent anti-tumour activity through irreversible devascularisation. Therapeutic synergy of ombrabulin with cisplatin has been shown in preclinical and phase I studies [48, 49]. In an industry-sponsored double-blinded phase III trial in pre-treated advanced STS of mixed subtype, patients were randomised to receive either ombrabulin (25 mg/m^2^ q3w) or placebo in addition to a chemotherapy backbone of cisplatin (75 mg/m^2^ q3w) [29]. After randomisation of 355 patients, a statistically significant improvement in PFS was seen in the ombrabulin-containing arm, but the absolute gain in median PFS of less than 1 week was deemed clinically insignificant (median PFS 1.54 vs 1.41 m, HR 0.76, 95% CI 0.59–0.98, *p* = 0.0302). No significant difference in overall survival was seen between study arms (median OS 11.4 vs 9.3 m, HR 0.85, 95% CI 0.67–1.09, *p* = 0.197). Response and clinical benefit rates were numerically higher in the ombrabulin arm (4 vs 1% and 47 vs 36%, respectively), as were rates of grade 3–4 neutropaenia (31 vs 19%). The small observed degree of incremental efficacy against a non-standard comparator arm has resulted in cessation of clinical development of ombrabulin in STS.

## Where did things go right? Where did things go wrong?

### Choice of endpoint

Overall survival, defined as time from randomisation to point of death from any cause, is the traditional gold standard endpoint in advanced STS phase III trials. Objective and precise, OS provides an unambiguously meaningful measure of treatment benefit. However, the large sample sizes and prolonged patient follow-up required for adequately powered OS analysis are a particular challenge in rare cancers. Furthermore, subsequent lines of post-trial therapy may confound the effect of any investigative agent on OS. Historically, first-line advanced STS drug trials were relatively shielded from these effects, given expected median overall survival of less than 1 year in control arms and few available further lines of therapy. However, recent first-line trials have reported median overall survival of 14.7–19.0 months in doxorubicin-only arms [3••, 4•, 26_•_, 30•]. This is a likely reflection of increasing trends for aggressive localised management of oligometastatic disease, a greater number of effective systemic therapies and improved access to specialist supportive and palliative care services. Longer follow-up is now required to measure OS in advanced STS, during which there is greater scope for confounding by post-trial treatment. It will be increasingly challenging for new treatments of advanced STS to demonstrate OS benefit, particularly where treatment effect is diluted by biological heterogeneity.

As clinical decisions to change treatment are typically based on evidence of disease progression, the use of endpoints based around progression events are of clear clinical meaning and are increasingly used in oncology trials as an OS surrogate. PFS, defined as the time from randomisation to disease progression or death, is not influenced by post-trial treatments, providing an earlier and increasingly clearer readout of treatment effect compared to OS. Whilst PFS is well validated in many tumour types and widely accepted by regulatory bodies as a legitimate efficacy measure, there remain limitations to its use as an endpoint in advanced STS. Little work has been performed to establish valid surrogacy of PFS for OS in STS. Assessment of disease progression is subjective and is vulnerable to bias in unblinded trials, potentially contributing to poor correlation between PFS and OS gains. Whilst PFS captures the benefit of cytostatic agents associated with low objective response rates, PFS can underestimate the benefit of treatments associated with unusual patterns of response, such as the cystic responses seen with some anti-angiogenic agents. Meanwhile, several recent STS drug trials have demonstrated large OS benefits in the absence of significant PFS improvement, calling into question the specificity of PFS as a surrogate for OS.

Patient quality of life (QoL) is an integral efficacy endpoint that can reflect changes in disease-related symptom burden, drug tolerability and psycho-social function of integral clinical meaning. Drug regulators are increasingly require supporting QoL data when considering new drugs for marketing authorisation, a trend reflected by the inclusion of QoL-related secondary endpoints in recent STS phase III trials. The expanded inclusion of QoL assessment to all phase III drug trials in STS should be matched by the continued development of valid and meaningful QoL measures specific to STS patients.

### Cohort selection

A majority of phase III drug trials performed in advanced STS over the past 30 years have recruited cohorts consisting of many different disease subtypes. A recent systematic review found that of 52 RCTs performed in STS between 1974 and 2014, only 7 of 52 (13%) included trials recruited from a single specified subtype [50]. Whilst permitting recruitment across a mix of STS subtypes increases the practicality of assembling sufficiently powered phase III cohorts, such ‘all-comer’ eligibility criteria introduces marked biological heterogeneity that may dictate widely varying sensitivity to an investigative treatment. It is therefore perhaps unsurprising that, in many cases, investigative treatments that have shown promising surrogate efficacy signals in small phase II studies fail to confer an aggregate survival advantage in larger, more heterogeneous phase III cohorts.

In the past 3 years, three large phase III trials have failed to demonstrate a survival advantage from combining an active alkylator with standard doxorubicin in the first-line treatment of similar ‘all-comers’ advanced STS cohorts [3••, 26•, 30•]. Across EORTC 62012, PICASSO III and SARC021, a strikingly consistent absolute increase of around 10% in objective response rates was seen with combination therapy, suggesting the presence of a disease subgroup with differential sensitivity. However, such a subgroup was not identifiable through analysis of histological or clinical factors in any of the studies, indicating some unidentified facet of disease that transcends established stratification factors. Meanwhile, this efficacy signal is diluted during follow-up of the entire cohort, resulting in the failure to translate into evidence of prolonged survival.

In contrast to these studies, the two phase III trials that have recently led to successful registration of eribulin and trabectedin both recruited from selected STS subtypes that had demonstrated sensitivity in earlier phase II studies [5, 6]. Similarly, low rates of 12-week progression-free survival in patients with adipocytic sarcomas treated in a phase II trial of pazopanib informed the exclusion of this subtype from the subsequent phase III trial that met its primary PFS outcome and led to the approval of pazopanib [11••, 51]. Whilst the timely recruitment of phase III cohorts consisting of a single or few STS subtypes has been considered impractical, recent studies have challenged this axiom by calling upon increasingly robust national and international collaborations [12••, 29]. The recently reported phase III trial of neoadjuvant chemotherapy performed by the Italian Sarcoma Group demonstrates the feasibility of completion of subtype-adaptive randomised protocols [52]. Accordingly, the focusing of phase III investigation of new drugs in pre-selected subtypes represents a viable and rational approach.

### Rapid progressors

In all recently reported STS phase III studies, between 25 and 50% of patients in both control and investigative arms experienced a progression event by the time of first protocol-mandated radiological assessment. With PFS curves superimposed to this point, in all summarised studies there is then a divergence of survival rates between control and investigative arms. In the cases of eribulin and trabectedin, the magnitude of effect of investigative agent was demonstrably sufficient to indicate a significant OS or PFS advantage. However, in PICASSO III and SARC021, the effect size of palifosfamide and evofosfamide was insufficient to demonstrate of significant difference after late separation of PFS curves.

Why do up to half of patients enrolled in advanced STS trials experience early progression, and can they be prospectively identified? The factors that contribute to early progression are likely numerous and variable. One possibility is that variation in assessment of performance status and life expectancy within large international, multi-centre studies results in the enrolment of patients already experiencing an irreversible terminal decline. Such a phenomenon is not however indicated by trial subgroup data, where little difference in treatment effect is reported between patients of PS 0 compared to those with PS 1 or 2. Similarly, any association between histopathological features and early progression would be expected to be revealed in subgroup analysis. It is plausible that currently uncharacterised aspects of disease biology are shared between rapidly progressing cases which, if identified, could be used to stratify trial populations as well as uncovering important disease drivers to serve as novel drug targets.

High rates of early progression remove significant proportions of trial cohorts from the at-risk population, thus diminishing the trial’s power to demonstrate any significant difference in outcome between patients who continue with protocol therapy beyond first radiological assessment. More research is required to understand why so many STS trial patients experience early progression and to develop means of prospectively identifying such patients in order to stratify study design and/or redirect poor prognosis patients away from futile therapies and toward supportive care measures.

## Where do we now go?

The full FDA approval of two new drugs in the past year represents an unprecedented rate of advance in the advanced STS armamentarium. However, the attrition rate of phase III trial to drug approval remains high and represents a disappointing output from significant investment of resources. Recent trial outcomes provide instruction for the design of future studies, all of which will rely upon ongoing national and international collaboration.

‘All-comer’ recruitment has been a common element of several recent negative STS phase III studies. A model of subtype preselection through use of surrogate progression-free and response endpoints in smaller phase II trials has proven to be more fruitful and should be followed in future drug development to reduce the biological heterogeneity within phase III cohorts. The development of tumour-based predictive and prognostic biomarkers should be at the forefront of all translational phase II and III endpoints to provide the means of cohort stratification and focusing of drug effect on sensitive biology. Reported tools of molecular stratification, such as the 67 gene CINSARC signature or gene expression-defined LMS subtypes, should be included in pre-specified subgroup analyses of trials in relevant STS subtypes [53, 54•].

Recent trial evidence suggests a new benchmark for estimated survival in doxorubicin control arms of first-line advanced STS trials. With the availability of up to six standard lines of systemic therapy for many STS subtypes, the use of OS as a primary endpoint in first-line advanced STS trials may now be unfeasible. Instead, wider use of PFS as a primary endpoint in this setting should be accompanied by greater effort to demonstrate definitive validity as a surrogate for OS, as well as the development of more sensitive and specific means for radiological assessment of response. The development of new imaging modalities that include dual energy CT, diffusion-weighted MRI and PET should be incorporated into the phase II and III development of new drugs in STS as a means of more accurate identification of sensitive subtypes. OS remains a viable secondary endpoint in trials in the pre-treated setting, but meticulous collection and reporting of post-trial treatments will be essential to establish the sources of any confounding or bias.

Finally, the broad availability of single patient-level baseline histological and clinical characteristics as well as efficacy data is essential for the detailed interpretation of STS phase III results. There is clear evidence across numerous drug trials that there are currently undefined patient subgroups that experience differential treatment responses and disease phenotypes.
